# Effect of pecto-intercostal fascial block on extubation time in patients undergoing cardiac surgery: A randomized controlled trial

**DOI:** 10.3389/fsurg.2023.1128691

**Published:** 2023-03-20

**Authors:** Lu Wang, Luyang Jiang, Ling Xin, Bailin Jiang, Yu Chen, Yi Feng

**Affiliations:** ^1^ Department of Anesthesiology, Peking University People's Hospital, Beijing, China; ^2^ Department of Cardiac Surgery, Peking University People’s Hospital, Beijing, China

**Keywords:** pecto-intercostal fascial block, tracheal extubation, cardiac surgery, pain, analgesia, postoperative

## Abstract

**Objectives:**

Epidural and paravertebral block reduce the extubation time in patients undergoing surgery under general anesthesia but are relatively contraindicated in heparinized patients due to the potential risk of hematoma. The Pecto-intercostal fascial block (PIFB) is an alternative in such patients.

**Methods:**

This is a single-center randomized controlled trial. Patients scheduled for elective open cardiac surgery were randomized at a 1:1 ratio to receive PIFB (30 ml 0.3% ropivacaine plus 2.5 mg dexamethasone on each side) or saline (30 ml normal saline on each side) after induction of general anesthesia. The primary outcome was extubation time after surgery. Secondary outcomes included opioid consumption during surgery, postoperative pain scores, adverse events related to opioids, and length of stay in the hospital.

**Results:**

A total of 50 patients (mean age: 61.8 years; 34 men) were randomized (25 in each group). The surgeries included sole coronary artery bypass grafting in 38 patients, sole valve surgery in three patients, and both procedures in the remaining nine patients. Cardiopulmonary bypass was used in 20 (40%) patients. The time to extubation was 9.4 ± 4.1 h in the PIFB group vs. 12.1 ± 4.6 h in the control group (*p* = 0.031). Opioid (sufentanil) consumption during surgery was 153.2 ± 48.3 and 199.4 ± 51.7 μg, respectively (*p* = 0.002). In comparison to the control group, the PIFB group had a lower pain score while coughing (1.45 ± 1.43 vs. 3.00 ± 1.71, *p* = 0.021) and a similar pain score at rest at 12 h after surgery. The two groups did not differ in the rate of adverse events.

**Conclusions:**

PIFB decreased the time to extubation in patients undergoing cardiac surgery.

**Trial Registration:**

This trial is registered at the Chinese Clinical Trial Registry (ChiCTR2100052743) on November 4, 2021.

## Introduction

Increased pain control and lower dosage of opioid agents with regional techniques are an important part of the enhanced recovery after surgery (ERAS) program in cardiac surgery (1). However, thoracic epidurals or paravertebral blockade are relatively contraindicated in heparinized patients due to the risk of hematoma, meanwhile, carrying underlying complications such as perioperative hypotension (2).

Sensory input from the chest wall mainly travels in the thoracic intercostal nerves; these nerves can be blocked by parasternal intercostal block, transverse thoracic muscle plane block (TTMPB), and pecto-intercostal fascial block (PIFB) (3). The parasternal intercostal block requires repeated punctures (4). The TTMPB has been shown to be efficacious in cardiac surgery (5, 6), but the transverse thoracic muscles are fairly thin and difficult to identify under ultrasound guidance. TTMPB is also prone to stimulate the pleura due to the relatively deep location of transverse thoracic muscles (7). PIFB is technically less challenging than TTMPB, and has been shown in a previous trial to possess comparable analgesic efficacy to the TTMPB in the area innervated by the anterior branches of the intercostal nerve (Th2-6), and thus useful in cardiac surgery (8). We conducted a single-center randomized controlled trial to test the hypothesis that PIFB facilitates early extubation by reducing intraoperative opioid consumption in patients undergoing cardiac surgery. Results are reported below.

## Methods and materials

This trial was conducted in the Peking University People's Hospital from 22 November 2021 to 18 March 2022. The trial protocol was approved by the Ethical Review Committee of Peking University People's Hospital (#2021PHB237-001) and is registered at the Chinese Clinical Trial Registry (www.chictr.org.cn, ChiCTR2100052743; November 4, 2021). Written informed consent was obtained from all participants.

## Participants

Adult patients (18 to 75 years of age) scheduled to undergo elective cardiac surgery with a full median sternotomy were eligible. Key exclusion criteria included: (1) known allergy to ropivacaine; (2) coagulation disorders; (3) infection at the puncture site; (4) psychical problems; and (5) a history of opioid abuse.

## Randomization, concealment, and blinding

Eligible patients were randomized at a 1:1 ratio to receive PIFB (30-ml 0.3% ropivacaine plus 2.5-mg dexamethasone on each side) or normal saline just after intubation before surgery. The randomization sequence was generated using a personal computer in a variable block size of 4 or 6. Concealment was conducted using opaque, sealed envelopes. Attending anesthesiologists, patients, and outcome assessors were blinded to the group allocation.

## PIFB and control procedure

**Figure 1:**
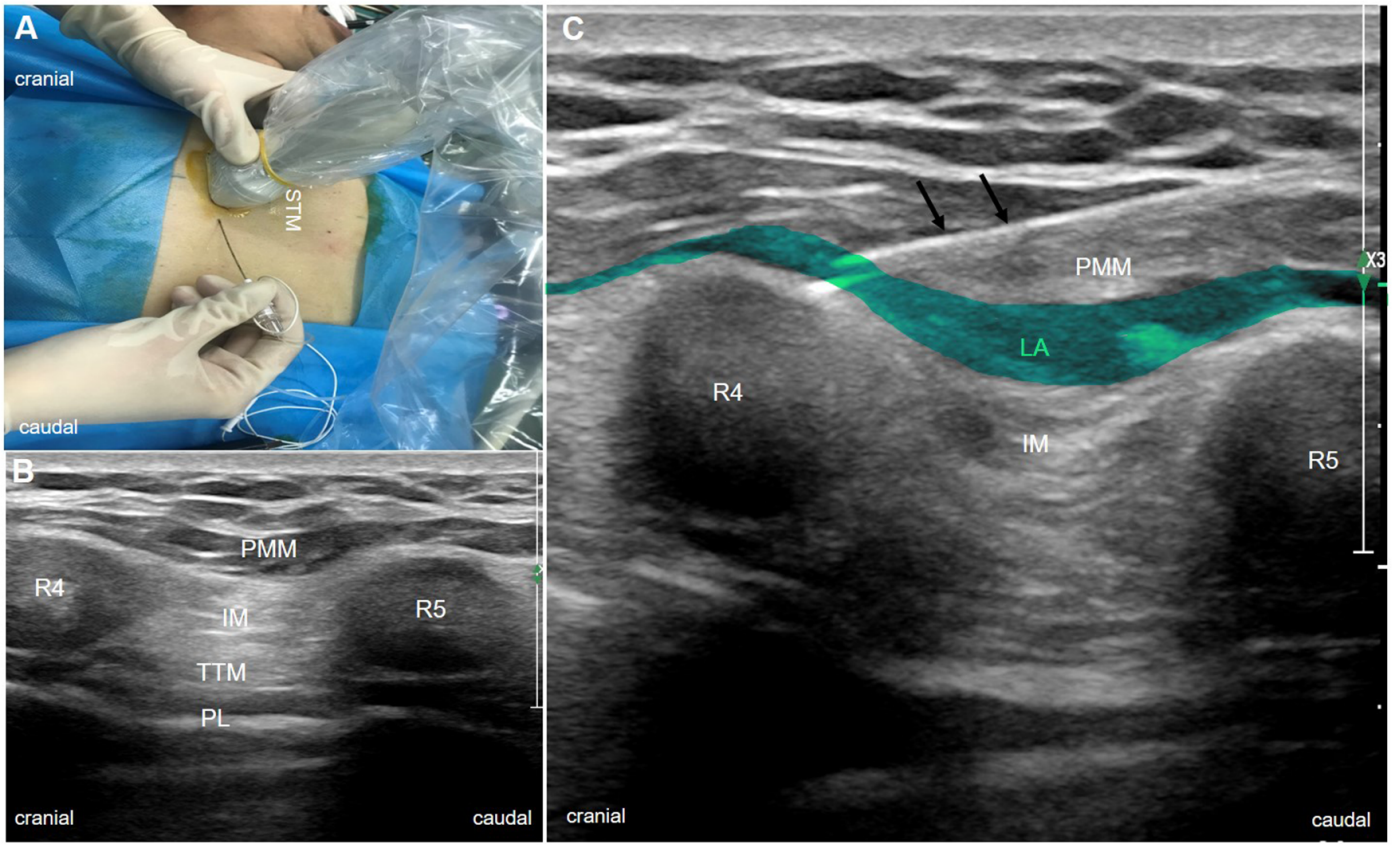
Procedures of PIFB. (**A**) patient positioning, longitudinal transducer, and needle orientation during PIFB. (**B**) anatomical location of PIFB on ultrasound (between the fourth and fifth ribs beside the sternum). (**C**) dissemination of local anesthetics with the movement of a needle (arrow). IM, intercostal muscle; LA, local anesthetics; PIFB, pecto-intercostal fascial block; PL, pleura; PMM, pectoralis major muscle; R4, fourth rib; R5; fifth rib; STM, sternum; and TTM, transversus thoracis muscle.

Bilateral PIFB was conducted in the supine position under ultrasound guidance after anesthesia induction. A high-frequency linear ultrasound probe (EPIQ7C, PHILIPS, Holland) was placed 2–3 cm lateral to the edge of the sternum to identify the pectoralis major muscle, intercostal muscle, internal thoracic vessels, and transversus thoracis muscle (Figure 1). Local anesthetics were injected into the fascial plane between the intercostal and pectoralis muscle, as previously described (9). A 21-gauge needle was inserted into the interfacial plane between the pectoralis major muscle and intercostal muscle at the fourth intercostal space using an in-plane technique. After verifying needle placement (visualizing the muscle separation upon injection of 2 ml saline), 30 ml 0.3% ropivacaine containing 2.5 mg dexamethasone was delivered to each side. Patients in the control group received 30 ml of normal saline.

## Anesthesia

All patients were sedated with midazolam (0.02–0.03 mg/kg). Anesthesia was induced with 0.2–0.4 mg/kg etomidate, 1–1.5 μg/kg sufentanil, and 0.2–0.3 mg/kg cisatracurium. Anesthesia maintenance was performed with propofol (100–200 mg/h), sevoflurane (0.8%–1.5%), dexmedetomidine (24–40 ug/h), and cisatracurium (6–10 mg/h) according to clinical needs to guarantee a bispectral index of approximately 45–55. Bolus sufentanil (0.3–0.5 μg/kg) was given upon an increase of blood pressure or heart rate by >10% over the baseline. Intravenous tropisetron (5 mg) was used to prevent postoperative nausea and vomiting (PONV) at the end of surgery. After surgery, patients were transferred to the intensive care unit (ICU) for further management.

## Postoperative analgesia

All patients received patient-controlled intravenous analgesia (PCIA) with a standard regimen of hydromorphone (no basal infusion, 0.2 mg bolus, and 10-minute lockout intervals). Postoperative pain was assessed using a 10-point numeric rating scale (NRS) (0 for no pain, 10 for the worst pain) at rest and upon coughing at 12, 24, and 48 h after surgery. A polypill consisting of oxycodone (5 mg) and acetaminophen (325 mg) was given as rescue analgesia for moderate to severe breakthrough pain for both groups. Intravenous tropisetron (5 mg) was used to treat PONV after surgery.

## Outcomes

The primary outcome was time to extubation (from completion of surgery to successful extubation). The timing of the extubation attempt was decided by the ICU staff using the following set of criteria: (1) fully awake with stable spontaneous ventilation; (2) stable hemodynamics with minimal inotropic support; and (3) warm peripheries. Secondary outcomes included intra-operative opioid consumption, hydromorphone consumption within 24 h, pain score at rest and upon coughing at 12, 24, and 48 h, moderate-to-severe pain (pain score of 4 or greater at any time within 48 h), time to drainage tube removal, and length of stay (LOS) in the ICU and hospital. Opioid-related adverse events included PONV, urinary retention, dizziness, and pruritus within 48 h.

## Statistical analysis

Sample size estimation was based on the following assumptions: (1) time to extubation of 12.4 ± 4.1 h in the control group and 9.0 ± 4.0 h in the PIFB group, based on a pilot trial that consisted of seven patients in each group; (2) *β* at 0.20 and *α* at 0.05; and (3) rate of 5%. The calculation yielded 50 subjects (25 in each group). The Kolmogorov-Smirnov test was used to assess the normality of continuous variables. Continuous variables other than pain score were analyzed using the Student's *t*-test. Pain score was analyzed using analysis of variance for repeated measures, with the Geisser-Greenhouse correction and Sidak's test for comparisons at each time point. Categorical variables were analyzed using Fisher's exact test. Statistical significance was defined as *p* < 0.05 (2-sided). All statistical analyses were performed using GraphPad Prism version 9.0.

## Results

**Figure 2:**
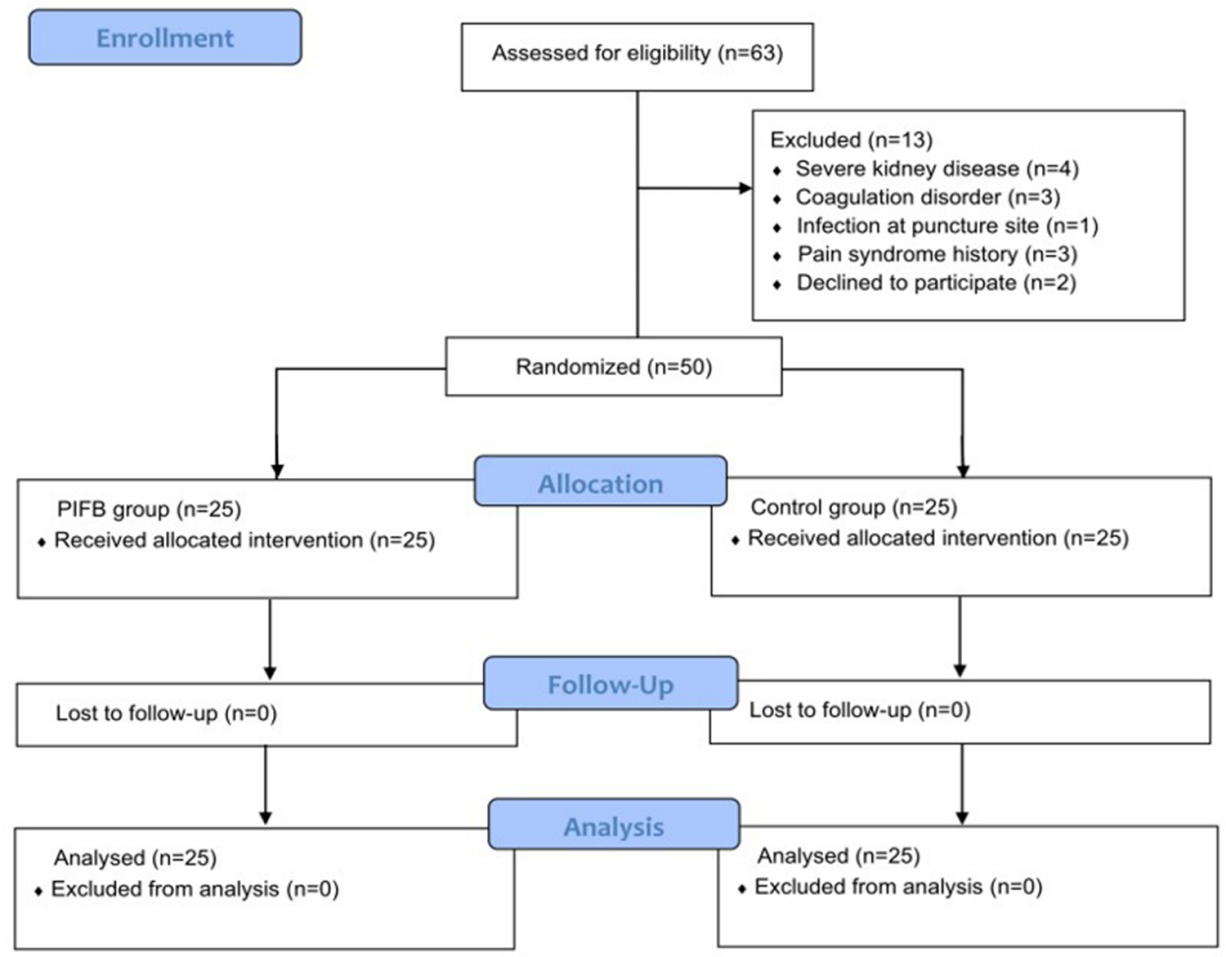
Patient flow through the trial.

A total of 63 patients were screened; 50 (mean age: 61.8 years; 34 men and 16 women) were randomized (25 in each group; Figure 2). The types of surgery included coronary artery bypass graft (CABG) in 38 patients, valve repair in three patients, and CABG plus valve repair in the remaining nine patients. The surgery was conducted under cardiopulmonary bypass (CPB) in 20 patients. The demographic and baseline characteristics of participants are shown in Table 1.

**Table 1:** Demographic and baseline characteristics.

	PIFB group (*n* = 25)	Control group (*n* = 25)	*P*
Age, mean ± SD, year	62.20 ± 12.86	61.40 ± 10.68	0.812
Men, *n* (%)	16 (64)	18 (72)	0.544
BMI, mean ± SD, kg/m^2^	24.92 ± 4.47	24.80 ± 3.62	0.917
**Comorbidities, *n* (%)**
Hypertension	15 (60)	18 (72)	0.370
Diabetes mellitus	6 (24)	8 (32)	0.529
Stroke	3 (12)	4 (16)	1.000
Chronic bronchitis	1 (4)	0 (0)	-
ASA, *n* (%)			1.000
II	1 (4)	2 (8)	
III	24 (96)	23 (92)	
NYHA, *n* (%)			0.765
II	16 (64)	17 (68)	
III	9 (36)	8 (32)	
**Surgery type, *n* (%)**
CABG alone	18 (72)	20 (80)	0.508
Valve surgery alone	2 (8)	1 (4)	1.000
Combined procedures	5 (20)	4 (16)	1.000
CPB, *n* (%)	10 (40)	10 (40)	1.000
EF before surgery, mean ± SD, %	65.0 ± 7.2	61.0 ± 9.2	0.089
E/A before surgery, median (IQR)	0.69 (0.63 to 0.82)	0.69 (0.52 to 0.83)	0.336
EuroSCORE II, median (IQR)	1.45 (0.98 to 3.15)	1.31 (0.85 to 2.82)	0.448
Surgical time, mean ± SD, min	284.4 ± 71.6	300.8 ± 66.3	0.406
Anesthetic time, mean ± SD, min	393.7 ± 75.7	408.3 ± 70.0	0.481

ASA, American Society of Anesthesiologists; BMI, body mass index; CABG, coronary artery bypass graft; CPB, cardiopulmonary bypass; EF, ejection fraction; EuroSCORE II, European System for Cardiac Operative Risk Evaluation II; IQR, interquartile range; NYHA, New York Heart Association; PIFB, pecto-intercostal fascial block; and SD, standard deviation.

Intubation and extubation were successful in the first attempt in all 50 patients. The time to extubation was 9.4 ± 4.1 h in the PIFB group vs. 12.1 ± 4.6 h in the control group (*p* = 0.031; Table 2). Total sufentanil consumption during the surgery was 153.2 ± 48.3 μg in the PIFB group vs. 199.4 ± 51.7 μg in the control group (*p* = 0.002). The PIFB and control groups did not differ in time to drainage removal (81.8 ± 23.7 vs. 76.9 ± 11.4 h, *p* = 0.350) and LOS in ICU (33.2 ± 24.7 vs. 37.0 ± 26.7 h, *p* = 0.604) or hospital (10.3 ± 5.3 vs. 10.1 ± 6.3 days, *p* = 0.904).

**Table 2:** Primary and secondary outcomes.

	PIFB group (*n* = 25)	Control group (*n* = 25)	*p*
Time to extubation, mean ± SD, hour	9.4 ± 4.1	12.1 ± 4.6	0.002*
Sufentanil consumption during surgery, mean ± SD, μg	153.2 ± 48.3	199.4 ± 51.7	0.031*
Hydromorphone consumption within 24 h, mean ± SD, mg	1.9 ± 1.3	2.1 ± 1.7	0.740
Moderate to severe pain, *n* (%)	5 (20)	10 (40)	0.123
Opioid-related adverse events, *n* (%)	11 (44)	9 (36)	0.564
Nausea and vomiting	5 (20)	2 (8)	0.415
Pruritus	0 (0)	0 (0)	-
Urinary retention	1 (4)	3 (12)	0.602
Dizziness	5 (20)	4 (16)	1.000
Length of stay in ICU, mean ± SD, hour	33.2 ± 24.7	37.0 ± 26.7	0.604
Time to drain removal, mean ± SD, hour	81.8 ± 23.7	76.9 ± 11.4	0.350
Length of hospital stay, mean ± SD, day	10.3 ± 5.3	10.1 ± 6.3	0.904

PIFB, pecto-intercostal fascial block; ICU, intensive care unit; and SD, standard deviation.

**Figure 3:**
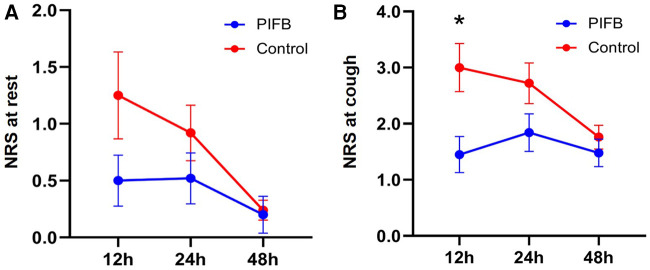
Postoperative pain score. (**A**) at rest. (**B**) upon coughing. Data were analyzed using 2-way ANOVA, followed by Sidak's multiple comparisons for each time point. Data are shown as mean ± standard error of the mean. *p < 0.05. NRS, numeric rating scale and PIFB,pecto-intercostal fascial block.

Pain scores at rest did not differ between the two groups (*p* = 0.137; Figure 3). Pain scores upon coughing were lower in the PIFB group than in the control group (*p* = 0.048). Post-hoc Sidak's test showed a lower pain score upon coughing at 12 h in the PIFB group (1.45 ± 1.43 vs. 3.00 ± 1.71 in the control; *p* = 0.021), and no significant difference at either 24 or 48 h. The two groups did not differ in the rate of moderate to severe pain (20% vs. 40%, *p* = 0.123), 24-h hydromorphone consumption (1.9 ± 1.3 mg in the PIFB group vs. 2.1 ± 1.7 mg in the control, *p* = 0.740), and opioid-related adverse events (44% vs. 36%, *p* = 0.564).

## Discussion

This trial demonstrated that PIFB performed before surgery can shorten extubation time in patients undergoing open cardiac surgery. Total opioid consumption during the surgery was lower in the PIFB group than in the control. PIFB also lowered pain score upon coughing at 12 h after surgery.

PIFB, as a novel fascial plane block, has been increasingly used in perioperative anesthesia and analgesia in non-cardiac surgery (10–12). Two randomized controlled trials have demonstrated that postoperative PIFB attenuates postoperative pain and reduces postoperative opioid consumption after cardiac surgery, but did not affect the duration of mechanical ventilation (13, 14). Such a finding was not surprising since PIFB was given after the surgery. Another trial reported decreased time to extubation by PIFB (15), but was not sufficiently powered since time to extubation was a secondary outcome.

The decrease in extubation time in the PIFB group in the current trial was approximately 3 h (from 12.1 ± 4.6 to 9.4 ± 4.1 h). Effect of such a magnitude is clinically relevant since early extubation in patients undergoing cardiac surgeries has been associated with improved oxygenation after cardiac surgery (16), and shorter stay in both the ICU and hospital (17, 18). In contrast, prolonged endotracheal intubation increases the incidence of dysphagia and postoperative pulmonary complications (19, 20).

Risk factors for a prolonged extubation time after cardiac surgery include older age, higher New York Heart Association (NYHA) class, and lower left ventricular ejection fraction (EF) (21). Reducing the use of anesthetics during cardiac surgery, particularly opioid agents, is a major strategy for promoting early extubation (22). Cardiac surgery with median sternotomy causes severe postoperative pain and requires very high doses of opioid agents, which in turn are associated with a series of opioid-related adverse events, e.g., respiratory depression and extended sedation. A previous trial demonstrated that PIFB when given prior to surgery could decrease the time to extubation, most likely by reducing intraoperative opioid consumption (15). Consistent with this notion, total intraoperative sufentanil consumption in the PIFB group was decreased by approximately 25% (from 199.4 ± 51.7 to 153.2 ± 48.3 μg) in our trial.

There was no difference in pain scores at rest between the two groups. Pain scores upon coughing were lower in the PIFB group than in the control group at 12 h (1.45 ± 1.43 vs. 3.00 ± 1.71 in the control; *p* = 0.021), and not significantly different at either 24 or 48 h. There was no difference in hydromorphone consumption within 24 h after surgery (1.9 ± 1.3 vs. 2.1 ± 1.7 mg, *p* = 0.740). The analgesic effects of PIFB lasted for 12 h after surgery in our trial. In patients requiring prolonged analgesic effects, continuous PIFB using an implanted catheter has been shown to provide analgesia over a period of 3 days (23). Physicians should balance analgesia and adverse effects to achieve better pain management (24). The two groups did not differ significantly in adverse events, including those related to opioid agents, within 48 h. LOS in the ICU and hospital also did not differ between the two groups. The lack of difference may be due to the relatively small sample size.

This trial has several limitations. First, the extubation time is decided by ICU staff using a set of subjective criteria, instead of a group of trained investigators. Such a design may introduce uncertainty to the data in a small trial even though ICU staff were unaware of the group allocation. Time to extubation exceeded 6 h even in the PIFB group. More attempts and practices may be needed to make the concept of early recovery after cardiac surgery accepted by clinicians. However, our findings indicated that the time to extubation was reduced. Second, PIFB was conducted after anesthesia induction (to maximize patient comfort). Accordingly, successful nerve blocks were uncertain in individual patients.

## Conclusions

PIFB decreased the time to extubation in patients undergoing open cardiac surgery, possibly due to lower requirements for intraoperative opioid consumption.

## Data Availability

The raw data supporting the conclusions of this article will be made available by the authors, without undue reservation.
